# Complete Genome Sequences of Mycobacteriophages Kwksand96 and Cane17

**DOI:** 10.1128/MRA.01182-18

**Published:** 2018-11-01

**Authors:** Kayla M. Fast, Tracy W. Keener, Shane Castleberry, Kenny D. Jones, John D. Larrimore, Caitlin A. Long, Jon T. Newburn, Nicholas C. Pritchett, P. Kiersten Schellhammer, Michael W. Sandel

**Affiliations:** a Department of Biological and Environmental Sciences, University of West Alabama, Livingston, Alabama, USA; Indiana University Bloomington

## Abstract

Bacteriophages Kwksand96 and Cane17 were isolated from Mycobacterium smegmatis mc^2^155. M. smegmatis is host to the highest number of phages analyzed from one species. Both mycobacteriophages were isolated from soil in west Alabama. Kwksand96 and Cane17 belong to subclusters B1 and C1, respectively, based on mycobacteriophage nucleotide sequence similarity.

## Abstract (precis)

Bacteriophages Kwksand96 and Cane17 were isolated from Mycobacterium smegmatis mc^2^155. M. smegmatis is host to the highest number of phages analyzed from one species.

## ANNOUNCEMENT

We report here the discovery and genome annotation of two mycobacteriophages from the Black Belt Geological Region of west Alabama. Cane17 was discovered on a farm in Emelle, Alabama (32.698678 N, 88.267897 W), and Kwksand96 was found on the University of West Alabama (UWA) campus (32.594337 N, 88.186615 W) in Livingston, Alabama. These phages were isolated and characterized by UWA students participating in the Science Education Alliance-Phage Hunters Advancing Genomics and Evolutionary Science (SEA-PHAGES) program.

The phages were isolated from the soil using the nonpathogenic host bacterium Mycobacterium smegmatis mc^2^155. The genus Mycobacterium also contains pathogenic species that cause tuberculosis, leprosy, and Buruli ulcer. Isolation of Kwksand96 was performed in 7H9 medium directly from the soil sample at 37°C. Conversely, 7H9 medium was enriched with the host bacterium when Cane17 was discovered. Imaging using a transmission electron microscope revealed that Kwksand96 is a *Siphoviridae* phage with a long tail; Cane17 possesses a relatively large head and short tail consistent with the *Myoviridae* family.

We extracted DNA from both phages using the Wizard DNA cleanup system (Promega, Madison, WI) in a modified protocol (1). Genomic DNA libraries were generated using an New England Biolabs (NEB) Ultra II FS kit (New England Biolabs, Ipswich, MA) with dual-indexed barcoding. Pooled libraries were run on an Illumina MiSeq system at the Pittsburgh Bacteriophage Institute at the University of Pittsburgh, which yielded at least 200,000 single-end 150-base reads for each genome. Read coverage depth was 861× and 188× for Kwksand96 and Cane17, respectively. We assembled these reads using Newbler v. 2.9 with default settings (2). Newbler produced a single-phage contig, which we checked for completeness, accuracy, and phage genomic termini with Consed v. 2.0 (3). No large buildups of reads or coverage variation were observed in either genome. We thus concluded that these genomes were circularly permuted. The first base of the genome was chosen to match similar, previously published phage sequences. Sequence data place Kwksand96 in the B1 subcluster and Cane17 in C1. Mycobacteriophage clusters were determined using whole-genome analysis, which places similar phages in the same clusters according to nucleotide sequence similarity and gene placement (4). The Kwksand96 genome is 68,882 bp long and has a G+C content of 66.4% and 103 protein-coding genes. The Cane17 genome is 160,330 bp long and has a G+C content of 64.7%, 223 protein-coding genes, 32 tRNAs, 1 transfer-messenger RNA (tmRNA), a programmed translational frameshift, and a wraparound gene (i.e., a gene that begins at the terminal end of the genome and wraps around to the beginning.).

Phage genomes were annotated in DNA Master v. 5.0.2 (http://cobamide2.bio.pitt.edu/). Starterator software was used to choose correct start codons for each gene (https://github.com/SEA-PHAGES/starterator). PhagesDB BLAST, NCBI BLAST, Phamerator (http://phamerator.org), and HHPred v. 3.0 were used to determine gene function (5–7). ARAGORN v. 1.2.38 and tRNAscan-SE v. 2.0 were used to detect and trim tRNAs (8, 9). Coding potential was determined with GeneMark v. 3.25 (10). We assigned functions to 25 of Kwksand96’s 103 protein-coding genes and 50 of Cane17’s 223.

### Data availability.

The complete genome sequences of Kwksand96 and Cane17 are available from GenBank under the accession numbers MH513973 and MH697579, respectively. Raw Illumina reads for Kwksand96 and Cane17 are available on NCBI’s Sequence Read Archive under accession numbers SRX4732908 and SRX4732909, respectively.
